# Supplementary material for: Development of a computer-aided design software for the quantitative evaluation of aesthetic damage
Source: PLoS One. 2019 Dec 18;14(12):e0226322. doi: 10.1371/journal.pone.0226322 (PMC6919621; doi:10.1371/journal.pone.0226322)
Supplement: S1 Code site — 1. (ZIP) [file pone.0226322.s001.zip › [EESC_jr][Projeto]Relatorio_07_21/reports/templates_zip/gentelella-master/vendors/Flot/examples/threshold/index.html]

Flot Examples: Thresholds


## Thresholds

With the threshold plugin, you can apply a specific color to the part of a data series below a threshold. This is can be useful for highlighting negative values, e.g. when displaying net results or what's in stock.

Threshold at 5
Threshold at 0
Threshold at -2.5

Copyright © 2007 - 2014 IOLA and Ole Laursen
